# The core competencies for mental, neurological, and substance use disorder care in sub-Saharan Africa

**DOI:** 10.3402/gha.v8.26682

**Published:** 2015-03-16

**Authors:** Pamela Y. Collins, Seggane Musisi, Seble Frehywot, Vikram Patel

**Affiliations:** 1 Office for Research on Disparities and Global Mental Health, National Institute of Mental Health, Bethesda, MD, USA; 2 Department of Psychiatry, Makerere University College of Health Sciences, Kampala, Uganda; 3 Department of Health Policy, The George Washington University, Washington, DC, USA; 4 Department of Global Health, The George Washington University, Washington, DC, USA; 5 Centre for Global Mental Health, London School of Hygiene & Tropical Medicine, London, UK; 6 Centre for Chronic Conditions and Injuries, Public Health Foundation of India, New Delhi, India

**Keywords:** mental health care, neurology, core competencies, human resources, task sharing, capacity-building, Africa

## Abstract

The 2010 Global Burden of Disease Study points to a changing landscape in which non-communicable diseases, such as mental, neurological, and substance use (MNS) disorders, account for an increasing proportion of premature mortality and disability globally. Despite evidence of the need for care, a remarkable deficit of providers for MNS disorder service delivery persists in sub-Saharan Africa. This critical workforce can be developed from a range of non-specialist and specialist health workers who have access to evidence-based interventions, whose roles, and the associated tasks, are articulated and clearly delineated, and who are equipped to master and maintain the competencies associated with providing MNS disorder care. In 2012, the Neuroscience Forum of the Institute of Medicine convened a meeting of key stakeholders in Kampala, Uganda, to discuss a set of candidate core competencies for the delivery of mental health and neurological care, focusing specifically on depression, psychosis, epilepsy, and alcohol use disorders. This article discusses the candidate core competencies for non-specialist health workers and the complexities of implementing core competencies in low- and middle-income country settings. Sub-Saharan Africa, however, has the potential to implement novel training initiatives through university networks and through structured processes that engage ministries of health. Finally, we outline challenges associated with implementing competencies in order to sustain a workforce capable of delivering quality services for people with MNS disorders.

The 2010 Global Burden of Disease Study points to a changing landscape in which chronic and non-communicable diseases account for an increasing proportion of premature mortality and disability globally (1). Mental, neurological, and substance use disorders (MNS) exemplify this trend: as a group they constitute the leading cause of disability worldwide (2). In sub-Saharan Africa, these are the most disabling disorders among people ages 10–44, with major depressive disorder and anxiety disorders ranking among the top five contributors (3).

Despite evidence of the need for care, a remarkable deficit of providers for MNS disorder services delivery persists in low- and middle-income countries (LMICs). In 2005, an estimated 1.18 million mental health providers were needed in LMICs (4). The projected annual cost of meeting these needs for the World Health Organization (WHO) Africa region is around US$207 million (4). Forecasts of mental health human resource demands in 2015 show few gains in resources (4), and forecasts for 2050 predict continued deficits compounded by increasing disease burden (5).

Beyond simply augmenting numbers, these providers must also be well-trained and mobilized in ways that use the existing health system infrastructure efficiently (4). One avenue to greater system-level efficiency is the thoughtful integration of mental health care into primary care and other chronic disease services (6–10). Chronic conditions, including MNS disorders, often require packages of care that include both psychosocial and pharmacologic interventions along with ongoing monitoring of health status (11). These similar approaches to care make integration feasible and collaborative models of care desirable. Thus, extending the assortment of health care workers able to deliver integrated care becomes essential. Task sharing, that is, the redistribution of tasks to health care workers with abbreviated training in the context of a team approach to care (12, 13), enables a range of local providers (e.g. trained community health workers, traditional healers, or nurses), with varied levels of specialization, to manage the care of multiple chronic conditions collaboratively, thus enlarging the mental health workforce.

A primary challenge in the implementation of services for MNS disorders is the ‘effective deployment of complementary skill sets in order to address the range of health problems’ (9). Implementation first requires consensus on the necessary interventions. For the global mental health context, the WHO Mental Health Gap Action Programme Intervention Guide (mhGAP-IG) is a resource that advocates specific treatments for the most prevalent MNS disorders (14). Additionally, management of MNS disorders requires accurate detection, care coordination, and continuity of services. Next, the tasks associated with each intervention must be delineated. In the example of treating a major depressive episode, a provider's task might be to deliver a psychological treatment. Finally, the consensus skill sets for the specified treatment intervention must be operationalized. Thus, a core set of skills (or competencies) must be articulated for each cadre of worker expected to collaborate in the delivery of MNS disorder services. These can then be the basis for the design and conduct of training and quality assurance interventions.

As in most countries, doctors and nurses in sub-Saharan African countries receive varying levels of training in the care for MNS disorders. In a sample of African countries for which WHO-Assessment Instrument for Mental Health Systems (WHO-AIMS) data are available, the percentage of hours in a nurse's training devoted to mental health ranged from around 1% to more than 20% (15–21). Doctors’ training showed similar wide variation by country, with a minority of countries delivering training for mental health comprising more than 10% of total training hours. Competency-based training is an approach that takes into account the specific health needs of a population, mapping these onto core sets of skills and establishing performance expectations for providers (22).

## The IOM workshop on competencies

In September 2012, the US Institute of Medicine's (IOM) Neuroscience Forum convened mental health and neurology researchers, providers, and advocates in Kampala, Uganda for a workshop that addressed the need to expand human resources for MNS disorder services. The workshop's goal was to convene ‘key stakeholders to discuss candidate core competencies that providers might need to help ensure the effective delivery of services for MNS disorders’ (23). Participants focused on candidate competencies for four MNS disorders that account for the greatest burden in LMICs: depression, psychosis, epilepsy, and alcohol use disorders.

Prior to the workshop the co-chairs (PYC, SM, and VP) and planning committee assisted the IOM on the development of a set of templates that outlined candidate core competencies for delivery of care by a range of providers for depression, psychosis, epilepsy, and alcohol use disorders. The provider categories for which competencies were identified were 1) community/lay workers (peers, community health workers, and health extension workers); 2) non-specialized non-prescribing practitioners (pharmacists, social workers, and occupational therapist); 3) non-specialized prescribing practitioners (clinical offers, nurses, and general medical doctors); and 4) specialized practitioners (psychiatric nurses, psychologists, neurologists, and psychiatrists). During the workshop, participants refined the candidate core competencies, which were divided into three categories: screening and identification, formal diagnosis and referral, and treatment and care.

## The core competencies for MNS disorders

The competencies corresponding to each provider category and disorder type can be viewed at the National Academies Press (http://www.nap.edu/catalog.php?record_id=18348). We present 24 shared ‘core’ competencies for all MNS disorder providers with relevance for the four prioritized disorders, across the three broad areas of intervention (Box 1). The competencies for these intervention domains focus on knowledge, comprehension, and application of the skills identified.

***Box 1*. Candidate core competencies discussed for all provider types across MNS disorders**

  Screening/identification (SI) 

SI.1. Demonstrates awareness of common signs and symptoms

SI.2. Recognizes the potential for risk to self and others

SI.3. Demonstrates basic knowledge of causes

SI.4. Provides the patient and community with awareness and/or education

SI.5. Demonstrates cultural competence

SI.6. Demonstrates knowledge of other mental, neurological, and substance use (MNS) disorders

  Formal diagnosis/referral (DR) 

DR.1. Demonstrates knowledge of when to refer to next level of care/other provider/specialist

DR.2. Demonstrates knowledge of providers for specialized care within the community

  Treatment/care (TC) 

TC.1. Provides support for patients and families while in treatment and care

TC.2. Identifies and assists patients and families in overcoming barriers to successful treatment and recovery (e.g. adherence, stigma, finances, accessibility, access to social support)

TC.3. Demonstrates ability to monitor mental status

TC.4. Demonstrates knowledge of how to offer emergency first aid

TC.5. Initiates and/or participates in community-based treatment, care and/or prevention programs

TC.6. Demonstrates knowledge of treatment and care resources in the community

TC.7. Promotes mental health literacy (e.g. to minimize impact of stigma and discrimination)

TC.8. Communicates to the public about MNS disorders

TC.9. Monitors for adherence to and/or side effects of medication

TC.10. Practices good therapeutic patient interactions (e.g. communication, relationship, attitude)

TC.11. Provides links between patients and community resources

TC.12. Identifies available resources to support patients (e.g. rehabilitation, medication supplies)

TC.13. Promotes activities that aim to raise awareness and improve the uptake of interventions and the use of services

TC.14. Protects patients and identifies vulnerabilities (e.g. human rights)

TC.15. Demonstrates respect, compassion, and responsiveness to patient needs

TC.16. Demonstrates knowledge and skills to use information technology to improve treatment and care

Note: This table is reproduced from the IOM meeting report, ‘Strengthening human resources through development of candidate core competencies for mental, neurological, and substance use disorders in sub-Saharan Africa: workshop summary’ (23).

### Screening/identification

Participants proposed six core competencies. All providers should be able to recognize the signs and symptoms of the priority disorders and demonstrate basic knowledge of the causes. They should demonstrate knowledge, that is, know that an assortment of disorders exist and be able to educate or make their communities aware of these disorders. Such community outreach requires that health workers achieve a level of cultural competence, the final core competency for this domain.

### Formal diagnosis/referral

Participants identified two competencies relevant to all providers: knowing when to refer and knowing what providers are available in the community to deliver specialized care.

### Treatment/care

Participants identified 16 core competencies which can be grouped into five categories: empathic communication (TC 10, 15), outreach and public awareness (TC 7, 8, 13, 14), linkages to community resources (TC 6, 11, 12), strengthening community care systems (TC 2, 5, 16), and direct care interventions for patients and families (TC 1, 3, 4, 9).

## Implementing and sustaining core competencies: building capacity and quality

The tasks and skill sets associated with each provider group will differ according to level of training and the context of the care setting. In lower income countries where the fewest specialist providers are available, prescribing professionals like clinical officers (a mid-level health worker with fewer years of training than a doctor) may perform tasks similar to those of a specialized practitioner in a middle-income country with more options for training and employing specialists (24). The developers of a competency-based curriculum for this type of provider would need to determine the subset of tasks and associated skills needed for the clinical officer based on the distribution of available health workers and the characteristics of the local population.

Ideally, training interventions will take into account provider characteristics (e.g. level of education, motivation for delivering services), the organizational context (e.g. peripheral clinic, district hospital, rural setting, existing mental health expertise), and characteristics of the patient population (e.g. pregnant women, people affected by the AIDS epidemic) (25). In addition, styles of teaching (e.g. active learning using role-play, coaching, and feedback) affect the likelihood of changing health workers’ behaviors. Tools to assess core mental health care competencies for use by non-specialist workers are now being developed in a number of settings and being used for evaluation of training and quality of psychological treatments (26).

Opportunities abound for testing the implementation of these core competencies by integrating them into curricular revision activities now in process at African universities that are establishing partnerships for medical and nursing training (27–30). Ministries of health and education play a critical role in implementation of competency-based learning, sometimes stimulating the reform of educational approaches, and by providing the necessary financial resources to carry it out (31). Implementation also requires working out institutional agreements and collaborations necessary to develop a competency-based training program of study; developing a realistic plan that includes establishing methods for distance learning and supervision, thereby ensuring access to adequate numbers of skilled professionals who can provide ongoing coaching and *in vivo* training that promotes the delivery of high-quality mental health care; providing credentialing and continuing education for professional development and maintenance of competencies; and establishing plans for monitoring and evaluation. E-learning technologies are being explored as a means for training nurses in Zambia and Ghana on mental health and maternal/child health (32) and are also being developed for alcohol and substance abuse care training (33).

Finally, successful implementation requires clinician educators to acknowledge the challenges of understanding, developing, and assessing competencies. Whereas competency-based training permits instructors to tailor training to the needs of the local context and to specify skills for mastery, detractors worry that it may be reductionistic, focusing on the minimum requirements – missing the whole in its focus on the specifics (34). Designers of competencies intended for international application must note that cultural context and translation to other languages may shift the meaning of competencies (34). Moreover, competencies required for a given provider shift over time, depending as much on local needs and human resource supply as on the political interests of professional societies and interprofessional relationships (35). Consequently, the assignment of competencies to particular cadres of providers is dynamic, and competencies may flow between provider types (35). We describe additional challenges associated with implementation of competencies in Box 2.

***Box 2*. Selected challenges and proposed solutions to implementation of core competencies in sub-Saharan Africa**

Challenges to Assessment of Competencies

- How to establish consistent high-quality assessment
  - Implement supervisor development programs to 1) train supervisors in behavioral observation, 2) ensure that supervisors understand what they need to assess and the criteria for assessment, 3) build consensus among trainers on specific behaviors that constitute an acceptable level of performance (36)
- How to assess the ‘whole’ of the trainee's performance rather than a series of fragmented behaviors
  - Note that qualitative and quantitative assessments can be applied
  - Utilize direct observation; subjective assessment can still be reliable (36)
- How to improve assessment overall
  - Incorporate assessment into daily routine
  - Research the implementation of assessment in a given context
  - Apply assessment in real life clinical or community settings
  - Ensure that assessments are aligned with actual tasks in the clinical or community context (36)

Challenges to Achieving and Maintaining Competent Performers

- ‘Incompetent’ clinical settings, i.e. settings in which healthcare providers are inadequately skilled facilitate the development of poorly trained providers (36, 37)
  - Establish partnerships with ‘Centers of Excellence’ to provide clinical mentoring for sites with under-skilled clinicians
- Dearth of available MNS disorder experts to supervise and assess health workers
  - Peer-led psychotherapy supervision, which has been shown to be acceptable to lay health providers, may provide an alternative to expert supervision in low-resource settings (26)
  - Peer assessors of therapy quality can achieve concordance with expert assessors (26)
  - Use technology to access remotely based experts for ongoing supervision and assessment
- Need for regular oversight of lay and professional providers to ensure that practice behaviors align with level of training and responsibility
  - Encourage and support regular communication among licensing/regulatory bodies related to different cadres of providers to ensure that provider-specific competencies remain relevant to the local context
  - Ensure that supervisory support for non-specialist providers reinforces referral pathways and measures to maintain safety for people with complex symptoms

Note: Challenges to assessment were adapted from Carraccio and Englander (36).

The time is right for implementation. The discourse on competence is rich and nuanced – able to inform training approaches in varied settings. The political context is ripe: governments are acting to accomplish the objectives of the WHO Comprehensive Mental Health Action Plan 2013–2020, which include ‘provide comprehensive, integrated, and responsive mental health and social care services in community-based settings’. The core competencies we describe provide a platform upon which context-specific curricula and training activities can be developed, launched, and evaluated.
